# Influence of age and fitness level on immune responses of T and NK cells in healthy physically active subjects after strenuous aerobic exercise: a cross-sectional study

**DOI:** 10.3389/fimmu.2023.1252506

**Published:** 2023-10-04

**Authors:** Paulo Francisco de Almeida-Neto, Ciro Alexandre Mercês Gonçalves, Phelipe Wilde, Jason R. Jaggers, Geraldo Barroso Cavalcanti Júnior, Valéria Soraya de Farias Sales, Radamés Maciel Vitor Medeiros, Paulo Moreira Silva Dantas, Breno Guilherme de Araújo Tinôco Cabral

**Affiliations:** ^1^ Health Sciences Center, Federal University of Rio Grande do Norte, Natal, Rio Grande do Norte, Brazil; ^2^ Department of Physical Education, Federal University of Rio Grande do Norte, Natal, Rio Grande do Norte, Brazil; ^3^ Department of Health and Sport Sciences, University of Louisville, Louisville, KY, United States; ^4^ Hemocentro Dalton Cunha - Hemonorte, Natal, Rio Grande do Norte, Brazil

**Keywords:** physical activity, immune system, leukocytes, lymphocytes, neutrophils

## Abstract

**Aim:**

The aim of this study is to analyze whether immune responses after strenuous exercise are influenced by chronological age and fitness level in physically active healthy men.

**Methods:**

Cross-sectional study with a sample of 32 physically active men. Participants were divided into two groups based on chronological age (younger: age 21.8 ± 1.8 vs. older: age 34.6 ± 8.3) and subsequently regrouped and divided based on fitness level (More conditioned: excellent and superior VO2max vs. Less conditioned: VO2max: weak, regular and good). Fitness was classified according to VO2max levels obtained by a treadmill test using a gas analyzer. Before and immediately after the ergospirometry test, blood samples were collected for evaluation of immunological markers: leukocytes, neutrophils, lymphocytes and subpopulations.

**Results:**

Chronological age had a moderate effect on CD3+CD4+ lymphocyte count (effect size: 0.204) and CD4/CD8 ratio (effect size: 0.278), favoring older subjects. The level of physical fitness had no significant effect on the analyzed immunological markers.

**Conclusions:**

Immune responses observed immediately after strenuous exercise may be more dependent on chronological age than on fitness level in healthy, physically active men.

## 1 Introduction

As people increase in age, a remodeling process of the innate and adaptive immune system occurs, called immunosenescence (i.e., aging and decline in efficiency of the immune system) (1). Such a process drives the body into a persistent pro-inflammatory state, and is responsible for increased susceptibility to infections, causing negative remodeling in leukocyte subpopulations (e.g., lymphocytes and neutrophils) (2) and occurring especially the loss of function in neutrophils (i.e., recognize and eliminate pathogens) and the change in the count of the major T-lymphocyte subpopulations (i.e., reduction of CD4+ and the elevation of CD8+, causing a decrease in the CD4/CD8 ratio) (3).

Living a healthy lifestyle is shown to be effective in reducing the damage of immunussenescence. The regular practice of physical activity is pointed out as a potential modulator of the immune system and can generate positive and negative adaptations depending on the intensity and volume of the training session (4, 5). Moderate exercise is known to be beneficial for the immune system, while strenuous exercise tends to promote negative responses that can lead to immunosuppression and an increased risk of upper respiratory tract infection (URTI) (6–9). Further, subjects with higher fitness levels, especially elite athletes, are apparently more resistant to negative immune system responses when compared to those sedentary individuals (10–13). However, it is not known whether in physically active individuals, immune responses present differently according to high or low fitness. Thus, the aim of the present study is centered on analyzing whether immunological responses after strenuous exercise are influenced by chronological age and fitness level.

## 2 Methods

**Figure 1:**
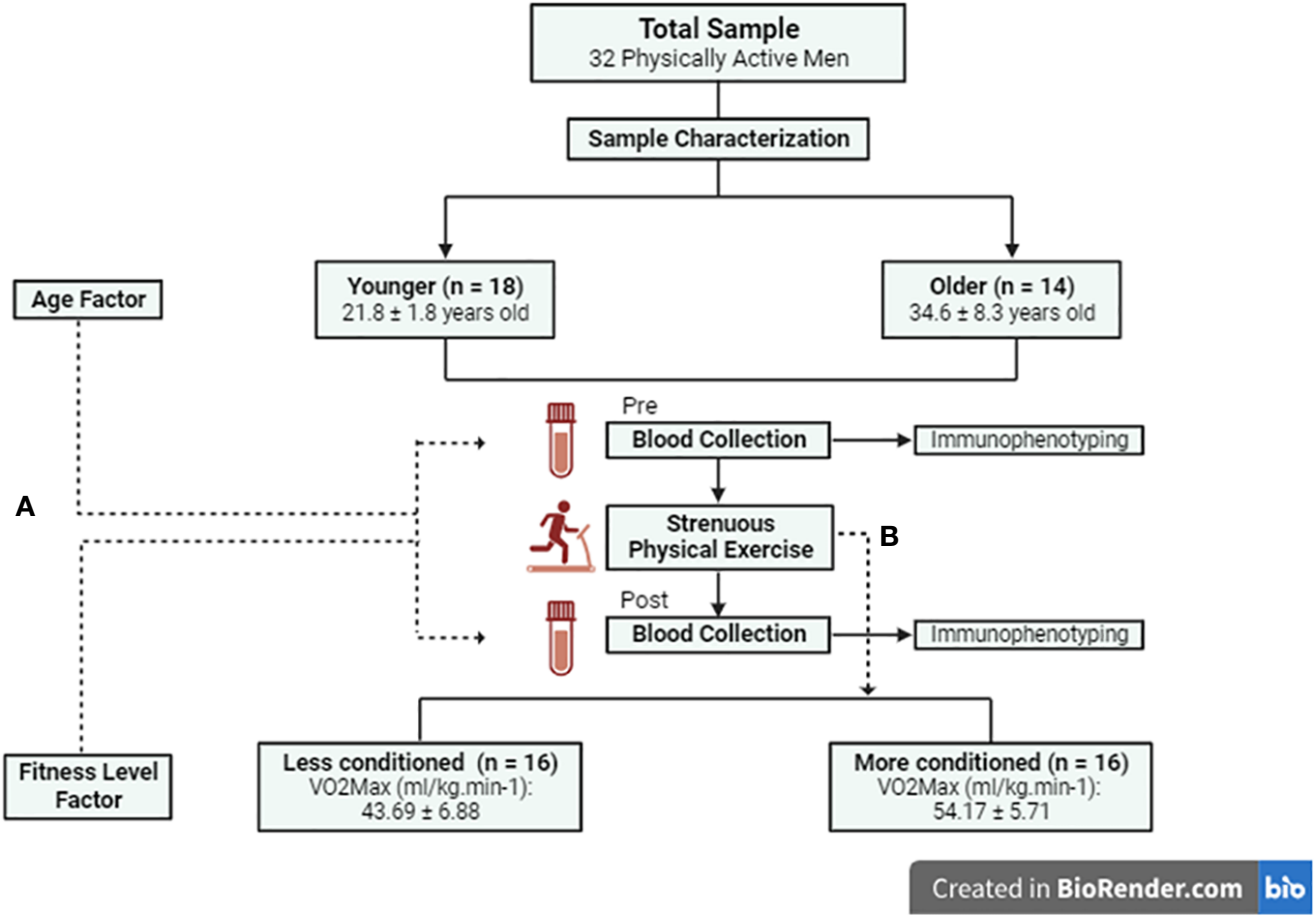
Study Flowchart. **(A)** Influence of age and physical fitness level on pre and post strenuous exercise immunological responses. **(B)** Fitness level groups were divided based on strenuous exercise.

A cross-sectional study, with a sample composed of 32 physically active men (**Figure 1**). We performed an *a priori* sample calculation, considering the effect size of η^2^p of 0.606. This size was found by Arroyo et al. (14), when analyzing CD4+ lymphocytes pre and immediately after high intensity physical exercise. Thus, with the help of the software G*Power (Version 3.1, Düsseldorf, Germany), considering the F statistic, an α = 0.05 and a standard β of 0.80, we reached a minimum sample size of 12 subjects per group (Effect size: 1.24, Critical F: 4.06, Power: 0.811).

The study was approved by the Research Ethics Committee of the Onofre Lopes University Hospital of the Federal University of Rio Grande do Norte (Natal, Brazil) (#1.252.034). All subjects participated voluntarily and signed an informed consent form in agreement with the ethical principles expressed in the World Medical Association’s Helsinki Declaration (15). The protocol of the present study was registered and is publicly available on the *Open Science Framework Registries* platform (Doi: 10.17605/OSF.IO/PRC6Z).

Participants were recruited through virtual social networks. The inclusion criteria were: (i) Being physically active for at least one year prior to the research; (ii) Being between 18 and 60 years old. Exclusion criteria were being a smoker, having clinically diagnosed heart problems, using hormonal or anabolic therapies that could interfere with the behavior of the immune system, and using immunomodulatory and/or immunosuppressive medication.

### 2.1 Procedures

For the blinding of the present study, the immunological indicators were analyzed by external collaborators who had no knowledge about the procedures of the present study. The researcher responsible for data collection and the participants had no knowledge of the classification of their fitness level. After the recruitment of the sample, 48-h before the tests, the volunteers were screened through a structured anamnesis. For characterization purposes, the volunteers who met the inclusion criteria completed a body composition analysis using dual-energy X-ray absorptiometry (DXA) and were instructed not to perform strenuous physical exercises in the 24 hours before the VO2max test.

### 2.2 Body composition assessment

Body weight was checked using a digital scale (Micheletti ®, São Paulo Brazil) with accuracy of 0.01kg. Height was measured using a stadiometer with an accuracy of 0.1cm (Sanny®, São Paulo, Brazil). Afterwards, the participants had their body composition assessed using a DXA (LUNAR®/GE PRODIGY - LNR 41.990, Washington, DC, USA) equipped with enCORE software (GE Healthcare®, version 15.0, Madison, WI, USA). The equipment was properly calibrated before the evaluations and followed the same configuration for all participants (Full Body Evaluation, Voltage (kV): 76.0, Current (mA): 0.150, Radiation dose (µGγ): 0.4 (Very low, no health risk)). Subsequently, the values in Kg of bone, fat, lean and fat-free mass were acquired.

### 2.3 Maximum cardiorespiratory capacity test

Because it is considered a strenuous physical exercise, the maximum cardiorespiratory capacity test was used in this study. The test was performed on a motorized treadmill (Centuriom 300®, Brasília, Distrito Federal, Brazil) located in an air-conditioned environment (24°C). Before the test a warm-up lasting5-min in duration at 4km/h and 0% inclination took place. During the test, heart rate was measured by short-range telemetry using a Polar® strap (Model H10, Vantage NV, Finland). The speed was increased gradually, according to the estimated capacity for each subject, based on the American College of Sports Medicine (ACSM), trying to reach the maximum oxygen consumption (VO2max) within the period of eight to twelve minutes. Details can be seen in the studies by Guazzi et al. (16), and Thompson (17). For the analysis of respiratory gases, an ergoespirometer Model Metalyzer-3B (Micromed®, São Paulo, Brazil) was used, following the “breath by breath” method. With the aid of Metasoft® software, connected to a Cortex® unit calibrated by the closed-circuit method with gas calibration, we determined the maximum oxygen consumption relative to body mass (ml/kg.min-1).

### 2.4 Subjective perception of effort

Was used the subjective rating of perception exertion (RPE) scale proposed by Borg (18) to measure the levels of physical effort perceived during the test, thus, the scale was exposed to the participant every 2 minutes. This scale consisted of numerical values between 6 and 20, where 6 indicates resting and 20 maximum effort. The incremental test lasted until the participant reported maximum effort on the Borg scale. We emphasize that there was previous familiarization with the RPE.

### 2.5 Blood samples

The peripheral blood collection (20mL) was performed by a nursing professional before and immediately after the maximal incremental test through the venipuncture method. Samples were collected using a vacuum vacutainer system (BECTON - DICKINSON - VACUTAINER SST BD), and 5mL were placed in tubes with containingethylenediaminetetraacetic acid or k3-EDTA anticoagulants (BD-Vacutainer, EDTA-K2 5.4 mg Plus Plastic) for blood count analysis (hematology analyzer, Cell Dyn-3.000®, São Paulo, Brazil), in which total leukocyte, lymphocyte and neutrophil counts were determined. For conversion to absolute values, the percentage values were multiplied by the absolute White Blood Cells (WBC) and divided by 100.

### 2.6 T-cell immunophenotyping

**Figure 2:**
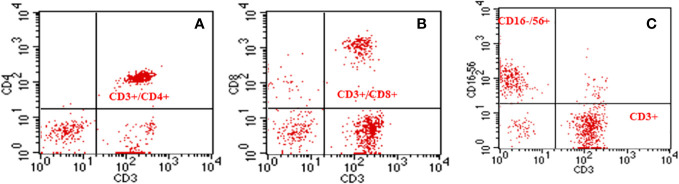
Graphical representation of dot-plot histograms obtained by flow cytometry of T-Helper, T-Cytotoxic and Natural Killer cell lymphocyte subpopulations. **(A)** Double labeling (CD3+/CD4+) showing the percentage of T-Helper T lymphocytes; **(B)** Double staining (CD3+/CD8+), showing the percentage of T- Cytotoxic lymphocytes; **(C)** Double staining (CD16-56+/CD3+), showing strong positivity for CD16/56 for Natural Killer (NK) Cells and CD3 for total T lymphocytes.

The lymphocyte subsets were analyzed using a lyse procedure based on a single-platform technique (19). The following 4-color combinations of MoAb were used to analyze antigen expression: fluorescein isothiocyanate (FITC), phycoerythrin (PE), peridin chlorophyll protein (PerCP) and phycoerythrin-cyanine (PC-5). Anti-coagulates whole blood (100μL each) was aliquoted in four 12 x 75 mm polystyrene tubes (Falcon Plastics, Becton Dickinson’s Biociences) containing 20 μL of each monoclonal antibody (MoAb). Tubes were mixed and incubated in the dark for 30 minutes (min) at room temperature. Furthermore, two mL of FACS Lysing Solution (Becton Dickinson’s Biociences), previously prepared in distilled water (1:10, v:v) was added to lyse the red blood cells. Tubes were agitated and incubates for 10 min, and centrifuged at 600g for 5 min in dark, the supernatant fluid was discarded, and the cell pellet was resuspended in cold phosphate buffered saline with Ph 7.2 (PBS, Signa-Aldrich, German) and centrifuged again. The last step was repeated. Finally, the cell pellet was resuspended in 1mL of 0.5% formaldehyde in PBS and cell suspension was kept in the dark at 4oC until flow cytometry analysis. A total of 20.000 events per tube were acquired with Fluorescence Activated Cell Analyzer (FACScan, San Jose, CA, USA) with Cell Quest software (Cell QuestTM® Software, Becton Dickinson Immunocytometry Systems, San Jose, CA, USA). In this way, identify lymphocyte subsets (CD3+, CD3+CD4+, CD3+CD8+, CD3+CD16+CD56+, CD16-CD56+, CD56+). Examples of immunophenotyping can be seen in **Figure 2**.

### 2.7 Fitness level factor

The fitness level factor was categorized based on VO2max levels following the recommendations proposed by Cooper (20), considering age and gender. The VO2max levels of the participants were classified as poor (n=3), fair (n=2), good (n=11), excellent (n=5) and superior (n=11). Given these results, we divided the sample into two groups:

1) Less conditioned (16 subjects. Group formed by participants with poor, fair and good VO2max ratings).
2) More conditioned (16 subjects. Group formed by participants with VO2max rating excellent and above).

### 2.8 Age factor

For analyses considering the age factor, we used the median split and divided the sample into two groups:

1) Younger (18 to 24 years old (n=18));
2) Older (26 to 59 years old (n=14)).

### 2.9 Statistical analysis

All data processing was performed in a blinded manner by a collaborator external to the present research. Descriptive data analysis was performed based on the recommendations of Mishra (21). To this end, the normality of the data of the independent variables (considering the time factor) were verified by the Shapiro-Wilk, Asymmetry and Kurtosis tests (-1.96 to 1.96). Levene’s test verified the assumption of homogeneity of variances for each independent variable (age & fitness level). In addition, we verified the sphericity of the data using Mauchly’s test. For the comparative analyses considering the factors time (Pre vs. Post), age (Younger vs. Older) and fitness level (Less conditioned vs. More conditioned), we used the Generalized Linear Model (GLM) followed by Bonferroni *post-hoc*. Thus, the effect size between the differences was checked by eta-squared-partial (η^2^p) considering the magnitude (22): Small<0.20; Medium>0.20 and <0.50; Large>0.50. For all analyses we considered a significance of p<0.05. SPSS Statistic software (IBM®, version 26.0, USA) was used for all analyses.

## 3 Results

The characteristics of the sample according to chronological age and fitness level are exposed in the **Table 1**.

**Table 1:** Sample characterization.

Variables	Chronological age			
	Younger (n = 18)		Older (n = 14)	
	Mean ± SD	Min; Máx	Mean ± SD	Min; Max
Age _(years)_	21.83 ± 1.85	18.00; 24.00	34.64 ± 8.38	26.00; 58.00
Height _(Cm)_	177.78 ± 7.97	160.00; 192.00	172.82 ± 5.47	165.00; 181.00
BMI _(Kg/cm²)_	24.44 ± 2.01	21.70; 28.22	26.55 ± 3.11	21.30; 32.00
Weight _(Kg)_	77.41 ± 9.64	61.60; 95.00	79.49 ± 11.43	58.00; 98.00
Bone mass _(Kg)_	3.47 ± 0.43	2.80; 4.54	3.10 ± 0.39	2.37; 3.89
Fat mass _(Kg)_	12.00 ± 2.97	6.93; 19.07	19.98 ± 5.95	8.83; 32.16
Lean mass _(Kg)_	61.45 ± 6.74	46.28; 72.20	56.51 ± 6.97	47.12; 68.96
Fat-free mass _(Kg)_	64.93 ± 7.10	49.13; 76.08	59.61 ± 7.30	49.80; 72.40
Resting heart rate _(pmb)_	68.33 ± 8.57	60.00; 80.00	60.00 ± 0.00	60.00; 60.00
Peak heart rate _(bmp)_	186.29 ± 28.23	126.00; 233.00	180.84 ± 13.93	160.00; 204.00
Maximum power _(Watts)_	854.60 ± 119.61	598.20; 1080.60	667.09 ± 115.00	381.30; 823.20
Relative anaerobic threshold 1 _(ml/kg.min-1)_	26.76 ± 5.43	15.43; 37.05	20.50 ± 6.00	9.55; 29.73
Relative anaerobic threshold 2 _(ml/kg.min-1)_	48.79 ± 7.18	31.70; 57.83	37.77 ± 7.34	28.08; 53.39
VO2 Max _(ml/kg.min-1)_	53.37 ± 6.03	38.38; 61.34	43.22 ± 7.06	33.76; 57.28
	Fitness Level			
	Less conditioned (n = 16)		More conditioned (n = 16)	
	Mean ± SD	Min; Máx	Mean ± SD	Min; Max
Age _(years)_	29.38 ± 7.08	19.00; 43.00	25.50 ± 9.62	18.00; 58.00
Height _(Cm)_	178.22 ± 6.72	165.00; 192.00	173.00 ± 7.15	160.00; 184.5
BMI _(Kg/cm²)_	26.48 ± 2.85	21.64; 32.00	24.24 ± 2.18	21.30; 28.22
Weight _(Kg)_	84.04 ± 9.42	65.90; 98.00	75.59 ± 7.89	58.00; 87.40
Bone mass _(Kg)_	3.43 ± 0.50	2.37; 4.54	3.19 ± 0.36	2.68; 3.92
Fat mass _(Kg)_	18.43 ± 6.24	10.28; 32.16	12.54 ± 4.09	6.93; 21.39
Lean mass _(Kg)_	61.40 ± 7.07	47.80; 72.20	57.19 ± 6.84	46.28; 70.79
Fat-free mass _(Kg)_	64.82 ± 7.51	50.17; 75.08	60.38 ± 7.15	49.13; 74.60
Resting heart rate _(pmb)_	62.50 ± 5.77	60.00; 80.00	66.88 ± 8.73	60.00; 80.00
Peak heart rate _(bmp)_	183;28 ± 21.08	137.00; 226.00	184.52 ± 25.34	126.00; 233.00
Maximum power _(Watts)_	734.95 ± 171.11	381.30; 1020.20	810.17 ± 117.646	598.20; 1080.60
Relative anaerobic threshold 1 _(ml/kg.min-1)_	22.17 ± 7.28	9.55; 32.80	25.87 ± 4.99	18.34; 37.05
Relative anaerobic threshold 2 _(ml/kg.min-1)_	38.84 ± 7.03	28.87; 53.27	49.09 ± 7.96	28.08; 57.83
VO2 Max _(ml/kg.min-1)_	43.69 ± 6.88	33.76; 57.76	54.17 ± 5.71	42.00; 62.34

BMI, Body mass index; (Kg/m²), Kilograms per square meter; (Kg), Kilograms; (bpm), Beats per minute; VO2 Max, Maximal oxygen uptake during maximal incremental test; (ml/kg.min^-1^), Millimeters per body weight per minute; SD, Standard Deviation; Min; Max, Minimum; Maximum.

The findings contained in **Table 2** indicate that for the leukocytes, lymphocyte and neutrophil count we found a significant effect for the time factor only. There was no significant effect for the fitness level. For the neutrophil percentage we found a effect of the age condition favoring older subjects, and we found no significant interactions between the time factor and the conditions (Fitness level & chronological age).

**Table 2:** Comparisons of leukocytes, neutrophil and lymphocyte counts considering the time factor (Pre & Post) and the Fitness level conditions (Less conditioned & More conditioned) and age (Younger & Older).

Variables	Condition	Pre	Post	General Linear Model*		
	Fitness level	Mean ± (SD)	Mean ± (SD)	Time	Condition	Interaction
Leukocytes _(mm³)_	Less conditioned	5643.75 ± 1484.57	10337.5 ± 1576.86 ¹	**< 0.001** (0.937)	0.096 (0.090)	0.877 (0.001)
	More conditioned	6693.75 ± 1508.85	11318.75 ± 2399.51 ¹			
Lymphocytes _(%)_	Less conditioned	40.06 ± 8.73	46.44 ± 10.83 ¹	**< 0.001** (0.489)	0.115 (0.081)	0.428 (0.021)
	More conditioned	34.38 ± 8.09	43.00 ± 7.49 ¹			
Lymphocytes _(mm³)_	Less conditioned	2209.13 ± 635.1	4760.88 ± 1143.23 ¹	**< 0.001** (0.832)	0.785 (0.003)	0.841 (0.001)
	More conditioned	2246.00 ± 493.01	4883.63 ± 1475.00 ¹			
Neutrophils _(%)_	Less conditioned	56.88 ± 8.48	44.58 ± 10.8 ¹	**< 0.001** (0.521)	0.062 (0.112)	0.974 (0.000)
	More conditioned	63.88 ± 6.12	50.26 ± 10.42 ¹			
Neutrophils _(mm³)_	Less conditioned	3.53 ± 1.11	7.01 ± 8.87 ¹	**< 0.001** (0.631)	0.239 (0.046)	0.201 (0.054)
	More conditioned	4.34 ± 1.18	5.93 ± 1.42 ¹			
	Age					
Leukocytes _(mm³)_	Younger	5905.56 ± 1079.47	10572.22 ± 1647.39 ¹	**< 0.001** (0.936)	0.342 (0.030)	0.970 (0.000)
	Older	6507.14 ± 2026.16	11157.14 ± 2519.68 ¹			
Lymphocytes _(%)_	Younger	39.22 ± 8.64	44.61 ± 9.10 ¹	**< 0.001** (0.524)	0.465 (0.018)	0.086 (0.095)
	Older	34.64 ± 8.55	44.86 ± 9.95 ¹			
Lymphocytes _(mm³)_	Younger	2279.22 ± 494.73	4666.28 ± 976.06 ¹	**< 0.001** (0.839)	0.685 (0.006)	0.268 (0.041)
	Older	2161.14 ± 646.67	5022.79 ± 1644.63 ¹			
Neutrophils _(%)_	Younger	58.23 ± 8.54	44.35 ± 12.32 ¹	**< 0.001** (0.514)	**0.042** (0.131)	0.288 (0.038)
	Older	63.14 ± 6.82	51.37 ± 7.17 ¹†			
Neutrophils _(mm³)_	Younger	3.64 ± 1.04	7.02 ± 8.31 ¹	**< 0.001** (0.620)	0.509 (0.015)	0.129 (0.075)
	Older	4.32 ± 1.31	5.76 ± 1.63 ¹			

(mm³), Cubic millimeters; (%), Percentual; (SD), Standard deviation. * General Linear Model (GLM) values are presented by p-value (η^2^_p_). ¹Significative difference (p < 0,05) between pre and post moments. †: Significative difference (p < 0,05) between younger and older groups during post-moment. The values in bold are those that present a statistically significant difference.

As shown in **Table 3**, we found an effect of time for the lymphocyte subpopulations regardless of the conditions (fitness level & age). However, for the age condition, we found no effect of time only for the variable CD56+. For the percentage of CD16-56+ we found a small interaction between the time factor and the fitness level condition. A moderate effect of chronological age was found for CD3+CD4+ levels and CD4/CD8 ratio favoring the older subjects in the prepartum period.

**Table 3:** Comparison of T-lymphocyte subpopulation levels considering time factor (Pre & Post) and Fitness level (Less conditioned & More conditioned) and chronological age (Younger & Older).

Variables	Condition	Pre	Post	General Linear Model*		
	Fitness level	Mean ± (SD)	Mean ± (SD)	Time	Condition	Interaction
CD3+CD16-56+ _(%)_	Less conditioned	3.13 ± 2.25	4.00 ± 3.45	0.094 (0.101)	0.123 (0.086)	0.947 (0.000)
	More conditioned	4.56 ± 3.31	5.69 ± 4.51			
CD3+CD16-56+ _(mm³)_	Less conditioned	66.46 ± 47.02	192.29 ± 175.28 ¹	**< 0.001** (0.705)	0.149 (0.076)	0.861 (0.001)
	More conditioned	104.89 ± 97.47	303.71 ± 316.78 ¹			
CD16-56+ _(%)_	Less conditioned	17.56 ± 10.89	36.56 ± 14.97 ¹‡	**< 0.001** (0.812)	0.174 (0.061)	**0.027** (0.152)
	More conditioned	10.38 ± 6.63	32.31 ± 11.49 ¹‡			
CD16-56+ _(mm³)_	Less conditioned	405.06 ± 330.29	1761.06 ± 952.44 ¹	**< 0.001** (0.898)	0.262 (0.042)	0.078 (0.100)
	More conditioned	248.29 ± 189.09	1526.8 ± 563.73 ¹			
CD56+ _(%)_	Less conditioned	0.00 ± 0.00	10.25 ± 22.46	**0.047** (0.125)	0.358 (0.028)	0.358 (0.028)
	More conditioned	0.00 ± 0.00	3.88 ± 15.5			
CD56+ _(mm³)_	Less conditioned	0.00 ± 0.00	220.51 ± 882.05	0.052 (0.121)	0.513 (0.014)	0.513 (0.014)
	More conditioned	0.00 ± 0.00	434.58 ± 944.42			
CD3+ _(%)_	Less conditioned	62.06 ± 9.36	48.63 ± 20.49 ¹	**< 0.001** (0.579)	0.085 (0.095)	0.548 (0.012)
	More conditioned	70.81 ± 7.99	54.56 ± 12.07 ¹			
CD3+ _(mm³)_	Less conditioned	1381.26 ± 459.12	2294.79 ± 1043.73 ¹	**< 0.001** (0.524)	0.225 (0.049)	0.522 (0.014)
	More conditioned	1580.45 ± 352.73	2726.36 ± 1294.98 ¹			
CD3+CD4+ _(%)_	Less conditioned	30.30 ± 7.50	23.88 ± 6.78 ¹‡	**< 0.001** (0.656)	0.141 (0.071)	**0.008** (0.213)
	More conditioned	39.88 ± 9.85	23.63 ± 7.37 ¹‡			
CD3+CD4+ _(mm³)_	Less conditioned	654.59 ± 203.46	1117.23 ± 388.38 ¹‡	**< 0.001** (0.477)	0.127 (0.076)	**0.038** (0.135)
	More conditioned	879.24 ± 231.82	1150.75 ± 567.27 ¹‡			
CD3+CD8+ _(%)_	Less conditioned	26.52 ± 6.35	24.38 ± 10.74	0.120 (0.079)	0.616 (0.008)	0.832 (0.002)
	More conditioned	28.44 ± 9.07	25.63 ± 12.31			
CD3+CD8+ _(mm³)_	Less conditioned	590.71 ± 229.77	1157.99 ± 598.41 ¹	**< 0.001** (0.661)	0.755 (0.003)	0.744 (0.004)
	More conditioned	644.01 ± 262.85	1331.47 ± 914.19 ¹			
CD4/CD8 _(%)_	Less conditioned	1.26 ± 0.62	1.14 ± 0.50 ¹	**0.009** (0.206)	0.565 (0.011)	0.134 (0.073)
	More conditioned	1.63 ± 0.91	1.25 ± 1.00			
	Age					
CD3+CD16-56+ _(%)_	Younger	4.17 ± 3.54	5.33 ± 5.03	0.092 (0.101)	0.829 (0.002)	0.866 (0.001)
	Older	3.43 ± 1.74	4.21 ± 2.26			
CD3+CD16-56+ _(mm³)_	Younger	95.05 ± 97.53	272.08 ± 321.18 ¹	**< 0.001** (0.710)	0.902 (0.001)	0.495 (0.017)
	Older	73.62 ± 41.28	217.04 ± 148.8 ¹			
CD16-56+ _(%)_	Younger	14.67 ± 9.10	34.11 ± 13.12 ¹	**< 0.001** (0.797)	0.672 (0.006)	0.205 (0.053)
	Older	13.07 ± 10.47	34.86 ± 14.01 ¹			
CD16-56+ _(mm³)_	Younger	337.09 ± 248.70	1583.63 ± 717.88 ¹	**< 0.001** (0.897)	0.640 (0.007)	0.106 (0.085)
	Older	313.29 ± 317.85	1721.46 ± 872.76 ¹			
CD56+ _(%)_	Younger	0.00 ± 0.00	12.56 ± 24.56	0.066 (0.108)	0.066 (0.108)	0.066 (0.108)
	Older	0.00 ± 0.00	0.00 ± 0.00			
CD56+ _(mm³)_	Younger	0.00 ± 0.00	582.30 ± 1156.71	0.070 (0.105)	0.070 (0.105)	0.070 (0.105)
	Older	0.00 ± 0.00	0.00 ± 0.00			
CD3+ _(%)_	Younger	66.72 ± 9.28	53.28 ± 19.71 ¹	**< 0.001** (0.582)	0.609 (0.009)	0.497 (0.015)
	Older	66.07 ± 10.43	49.43 ± 12.54 ¹			
CD3+ _(mm³)_	Younger	1524.35 ± 404.30	2483.17 ± 1062.01 ¹	**< 0.001** (0.523)	0.945 (0.000)	0.658 (0.007)
	Older	1424.93 ± 437.55	2545.81 ± 1352.50 ¹			
CD3+CD4+ _(%)_	Younger	30.26 ± 8.08	22.72 ± 7.41 ¹	**< 0.001** (0.639)	**0.009** (0.204)	0.070 (0.105)
	Older	41.29 ± 8.62 ^#^	25.07 ± 6.37 ¹			
CD3+CD4+ _(mm³)_	Younger	684.52 ± 212.21	1039.51 ± 360.74 ¹	**< 0.001** (0.432)	**0.046** (0.125)	0.596 (0.009)
	Older	872.84 ± 245.88 ^#^	1255.46 ± 589.59 ¹			
CD3+CD8+ _(%)_	Younger	30.41 ± 6.55	27.78 ± 11.7 ¹	0.127 (0.076)	**0.034** (0.142)	0.913 (0.000)
	Older	23.71 ± 7.77 ^#^	21.43 ± 10.27 ¹			
CD3+CD8+ _(mm³)_	Younger	695.88 ± 231.89	1328.99 ± 741.79 ¹	**< 0.001** (0.667)	0.077 (0.101)	0.460 (0.018)
	Older	516.41 ± 229.25	1136.4 ± 808.69 ¹			
CD4/CD8 _(%)_	Younger	1.04 ± 0.38	0.97 ± 0.51	**0.008** (0.215)	**0.002** (0.278)	0.260 (0.042)
	Older	1.96 ± 0.89 ^#^	1.49 ± 0.98 ¹			

(mm³), Cubic millimeters; (%), Percentual; (SD), Standard deviation. * General Linear Model (GLM) values are presented by p-value (η^2^_p_). ¹ Significative difference (p < 0,05) between pre and post moments. ‡: Significant interaction (p<0.05) between the factors time and fitness level. ^#^ Significative difference (p < 0,05) between younger and older groups during pre-moment. The values in bold are those that present a statistically significant difference.

## 4 Discussion

The present study aimed to analyze whether immune responses after strenuous exercise were influenced by chronological age and fitness level. Our initial hypothesis was that more conditioned and/or younger subjects would be less susceptible to negative changes in immune responses compared to their peers. The results of the present study indicated that in the sample analyzed, the immune responses were not significantly dependent on fitness level; however, there was a significant effect of chronological age on the CD3+CD4+ and CD4/CD8 ratio. In order to facilitate the discussion of results, we will divide the discussion between chronological age and fitness level.

### 4.1 Advancing chronological age & immune responses

Aging is closely linked to reduced efficiency of the immune system (23). This may be related to the thymus gland (i.e., primary lymphoid organ), which begins the involution process after puberty (24, 25). Lawton (26) highlights that thymic involution occurs at a rate of approximately 3% per year. According to Miller (27), the main function of the thymus is to provide the mechanisms for T-cell maturation. In a review study, Yan et al. (28), indicated that advancing thymic involution process reduces the efficiency of the gland with respect to promoting T-cell maturation, which generates decay of the lymphocyte population. According to Wikby et al. (29), a low proliferative response of lymphocytes or the ratio of CD4/CD8 T cells is closely related to aging, pointing lower values in subjects with advanced chronological age. This may justify the findings of the present study regarding the effect of the chronological age factor on CD3+CD4+ and CD3+CD8+ lymphocytes and the CD4/CD8 ratio.

Jacome Burbano, Cherfils-Vicini & Gilson (30) emphasize that among the immune cells that suffer the greatest effect of aging are neutrophils. Such cells are part of the innate immune system and act as the body’s first line of defense against infection (31). According to Petri & Sanz (32), neutrophils migrate to extravascular sites of infection or tissue damage through a process called chemotaxis, and this process is what becomes most defective as the immune system ages. Thus reducing the speed and efficiency of neutrophils, and can cause increased low-level generalized inflammation (33). According to Bartlett et al. (10), one way to restore neutrophils and improve their function is to engage in physical activity on a regular basis. The fact that the sample in the present study was physically active may justify the findings on the small effect of chronological age on neutrophil percentage.

Lawton (26) reports that healthy lifestyle habits (e.g., organic food and exercise) can rejuvenate the immune system and may increase its efficiency. In addition, he points out that in general, the immune system has a biological age rhythm that may differ from chronological age, and this concept is called immune age. Thus, it is possible for younger subjects to have an immune age like that of an older subject, and the reverse is also true. In this sense, because our sample consisted of healthy physically active subjects, it is possible to suggest that the immune age is balanced between the groups (Younger & Older). This would be a possible explanation as to why the present study found no significant effect of chronological age on the other immunological variables analyzed.

### 4.2 Physical conditioning & immune responses

The influence of exercise intensity and fitness on immune responses gained prominence with the development of the famous “J-curve” (9). A consensus that currently exists is that regular moderate-intensity physical activity entails greater immunovigilance when compared to sedentary individuals (34). In addition, fitness level, light to moderate physical activity, and even performing exercise before or after an influenza or COVID-19 vaccination appears to increase the antibody response to vaccination (35, 36).

Regarding the comparison of immune responses in physically active individuals after performing a maximal exercise test, there seems to be no difference when compared in groups according to fitness levels. The results of the present study did not show a statistically significant difference between individuals with superior or excellent fitness compared to those with good, fair or poor fitness in any of the immune markers, except for CD3+CD4+ (% and mm3) and CD16-56+ (%) which showed statistically significant differences. To the best of our knowledge, this was the first study to compare immune responses after a maximal exercise session in physically active individuals divided into groups based on current fitness level.

However, according to the study conducted by Dorneles et al. (37), fitness status seems to directly affect T-lymphocyte function, i.e., those individuals with lower fitness have higher circulating T-lymphocytes and a consequent higher pro-inflammatory state. Furthermore, in another study conducted by Dorneles et al. (38), it was identified that after three sessions of high-intensity interval training (HIIT) with 48h of recovery between sessions, low fitness exerted a negative impact on several immune system markers. Thus, although fitness level is shown to play a key role on the immune system, there appear to be no differences in immune responses immediately after a single maximal exercise session, however, it is not known how this immune response behaves in the following hours at different fitness levels.

### 4.3 Limitations and suggestions for further studies

The main limitations of the present study were: (i) not having a group of sedentary subjects, which could have provided us with more robust answers regarding the effect of fitness level on immunological factors after strenuous exercise and dividing groups by each fitness level, and not only into higher and lower; (ii) The group of less-conditioned subjects was composed mostly of subjects with good fitness levels; (iii) not having a group composed predominantly of pediatric, middle-aged, or elderly subjects, which would allow us more precise findings on the effect of advanced chronological age on the immune system after performing strenuous exercise; (iv) not having performed more extended analyses over time (e. g., after 24 h of the exercise session) and assessments of the risk of upper respiratory tract infection. We suggest that future studies conduct investigations like the present study considering sedentary subjects, fitness levels divided by all categories and different age groups, as well as differences between male and female sexes.

### 4.4 Practical applicability

The main finding of the present study was that for physically active adult men, strenuous exercise will generate similar immune responses immediately after, regardless of fitness level. Thus, in physically active men, fitness level is apparently not a protective factor for negative immune system responses immediately following strenuous exercise. Thus, sports professionals and fitness coaches cannot overload subjects with high fitness levels on the assumption that they will be less susceptible to the negative effects of strenuous exercise on immunity. Such care will optimize recovery between strenuous physical training sessions and may prevent possible occurrences of overreaching and/or overtraining.

## 5 Conclusion

The results allow us to conclude that in healthy physically active men, the immune responses immediately after strenuous exercise seem to depend not on the level of physical conditioning but on chronological age. These results are clear when they show that advancing chronological age influences the reduction of the neutrophil percentage and on the reduction of the CD4/CD8, CD3+CD8+ and CD3+CD4+ ratio in older subjects.

## Data availability statement

The data for this study is publicly available at: https://figshare.com, under the Doi: 10.6084/m9.figshare.22364611.

## Ethics statement

The studies involving humans were approved by Research Ethics Committee of the Onofre Lopes University Hospital of the Federal University of Rio Grande do Norte. The studies were conducted in accordance with the local legislation and institutional requirements. The participants provided their written informed consent to participate in this study.

## Author contributions

PdA-N and PW: Conception of the initial idea, application of the study protocols, interpretation of results, translation, writing and final validation of the article. CG and JJ: Writing, grammatical correction and final validation of the article. GJ and VdF: Analysis of immunity indicators, writing and final validation of the article. RM: Analysis of the statistical data, writing, grammatical correction and final validation of the article. PD: Project supervision, data analysis/interpretation, and drafting of the article. BdATC: Concept/design, project supervision, data collection, drafting of the article, and critical revision of the article. All authors contributed to the article and approved the submitted version.
